# Mineral and Carbon Metabolic Adjustments in Nodules of Symbiotically Grown Faba Bean (*Vicia faba* L.) Varieties in Response to Organic Phosphorus Supplementation

**DOI:** 10.3390/plants12223888

**Published:** 2023-11-17

**Authors:** Frank K. Amoako, Saad Sulieman, Karl H. Mühling

**Affiliations:** Institute of Plant Nutrition and Soil Science, Kiel University, Hermann-Rodewald-Straße 2, 24118 Kiel, Germany; fkamoako@plantnutrition.uni-kiel.de (F.K.A.); ssulieman@plantnutrition.uni-kiel.de (S.S.)

**Keywords:** *Vicia faba*, biological nitrogen fixation, dinitrogen, metabolites, organic phosphorus, minerals, rhizobia

## Abstract

Phosphorus (P) is a major limiting factor for legume and symbiotic nitrogen fixation (SNF). Although overall adaptations of legumes to P supplementation have been extensively studied in connection with inorganic P, little information is currently available regarding nodulation or SNF responses to organic P (Po) in hydroponics. We investigated the mineral and carbon metabolism of Po-induced nodules of two contrasting faba bean varieties grown hydroponically under inorganic P (Pi), viz., in P-deficient (2 µM KH_2_PO_4,_ −Pi), sufficient-P (200 µM KH_2_PO_4_, +Pi), and phytic acid (200 µM, Po) conditions, and were inoculated with *Rhizobium leguminosarum* bv. *viciae* 3841 and grown for 30 days. The results consistently reveal similar growth and biomass partitioning patterns between +Pi and Po, with both varying substantially from −Pi. In comparison, +Pi and Po observed equivalent accumulations of overall elemental P concentrations, with both increasing by 114 and 119%, respectively, relative to −Pi. A principal component analysis on metabolites showed a clear separation of the −Pi treatment from the others, with +Pi and Po correlating closely together, highlighting the nonsignificant differences between them. Additionally, the δ^15^N abundance of shoots, roots, and nodules was not significantly different between treatments and varieties and exhibited negative δ^15^N signatures for all tissues. Our study provides a novel perspective on mineral and carbon metabolism and their regulation of the growth, functioning, and reprogramming of nodules upon phytate supply.

## 1. Introduction

Globally, the heavy use of chemical nitrogen (N) fertilizers in agriculture is costly in economic terms, is harmful to the environment, and is known to compromise soil health [1]. Sustainability demands a paradigm shift in this phenomenon, and the proposed solution is biological nitrogen fixation (BNF). BNF is the microbiological process of converting atmospheric nitrogen into plant-usable forms and synthesizing biomolecules useful in plants [1]. BNF offers several benefits over nitrogen fertilizers (N), including more efficient use of N by plants, reduced N leaching, and a healthier and cleaner environment [2]. Furthermore, BNF has positive effects on crop rotation and non-legumes in the mixed cropping system and is known for sustaining high productivity in agriculture [2,3].

However, the process of symbiotic nitrogen fixation (SNF) in dinitrogen (N_2_)-fixing legumes is hampered by a plethora of abiotic stressors [4,5] such as nutrient deficiencies. Consequently, SNF and nodule formation and functioning in the legume–rhizobia symbiotic relationship has a substantial demand for mineral elements (especially P) for plant and bacterial growth, metabolic regulation, and operations like synthesizing macromolecules. For instance, P is required in an optimum amount for the biosynthesis of ATP needed for powering the nitrogenase enzyme in bacteroid [6]. However, the primary source of P for this process has been solely inorganic P (Pi). It is, however, not surprising that several studies have determined the detrimental effects of P on SNF, focusing exclusively on Pi in their experiments [7,8,9,10,11,12,13], with the complete neglect and exclusion of the organic P (Po), which is the largest fraction in soil.

Over the last decade, numerous researchers have demonstrated and established that multiple plant species have the potential to acquire and utilize Po in soil [14,15,16,17,18,19] and hydroponics systems [20]. However, the effect of Po on legume–rhizobia growth and metabolism, such as the synthesis of macromolecules and nitrogenase enzymes, remains inexplicable. The Po has been evident as a competent alternative to the ever-predicted-to-be-exhausted Pi. Our group, however, was prompted by this development to research and gain insight into the effects of Po on SNF and nodulation in soil and hydroponics systems. Previously in our group, we have demonstrated that different faba bean varieties can acquire and utilize various sources of Po efficiently in soil [21,22]. These studies showed that faba bean plants supplemented with different Po sources produce equal biomass and have nodulation and SNF capacity similar to Pi. A recent Game Changer documentary [17] has drawn attention to the proponent that symbiotic legumes can mobilize soil Po resources competently. However, there is not much direct information regarding the potential mechanisms underlying the differential Po uptake and metabolism by nodulating plants in hydroponics systems. These goals can be accomplished not only by the dynamics of soil organically bound phosphorus, but also by exploring the various mineral and carbon metabolic regulatory pathways of the symbiotic nodules at the whole-plant level. These advances can significantly assist in preserving the limited inorganic phosphorus (Pi) resources and minimizing the wasteful applications of chemical Pi fertilizers.

Several signaling molecules have been hypothesized to be involved in the interaction between the host and the symbiotic tissue based on the structural differences in the nodule anatomy and metabolism among leguminous species, leading to the regulation of genes, enzymes, and metabolites [12]. Compelling efforts to ascertain Po’s synergistic and antagonistic interactions with these signaling biomolecules and mineral nutrients in faba bean under symbiotic conditions would be auspicious and rewarding in the future. Furthermore, previous studies have provided mesmerizing evidence that the ability of plants to use P from Po is markedly improved when they are inoculated with the ideal soil microorganisms [21]. Soil microorganisms (inoculants) that possess phosphatases specific for the dephosphorylation of phytate have been reported to enhance Po uptake [16]. Although many investigations have been carried out and have noted the ramifications Po imposes [15,19] on growth, biomass accumulation, utilization, and acquisition efficiencies of plants, it is still unknown how nodulated faba bean plants precisely respond to Po assimilation at the metabolite level under symbiotic conditions. By understanding the complex strategies by which legumes respond to Po nutrition, we can develop artificial symbiotic plants via breeding with enhanced symbiotic capacity. We can biotechnologically develop more efficient plants for Po acquisition and utilization. The development of Po-efficient crop plants that can grow and yield better would significantly impact agricultural sustainability and the quest to find a suitable alternative to Pi. 

Thus, in the present study, we investigate how Po substrates (i.e., inositol phosphates) coordinately facilitate the mobilization, nutrient uptake, and anion and carbon metabolism in nodules of faba bean varieties that differ in organic phosphorus use efficiency (PoUE), as previously described [22], and assess the effect of Po on the symbiotic capacity and nitrogen fixation of *Vicia faba* plants when exclusively relying on N derived from nodule symbiosis. The present study hypothesized that higher P contents induced by phytic acid supplementation might play a significant role in the carbon metabolism by improving and providing the minerals and carbon required for the functioning of nodules.

## 2. Results

### 2.1. Growth and Dry Matter Accumulation and Allocation

**Figure 1:**
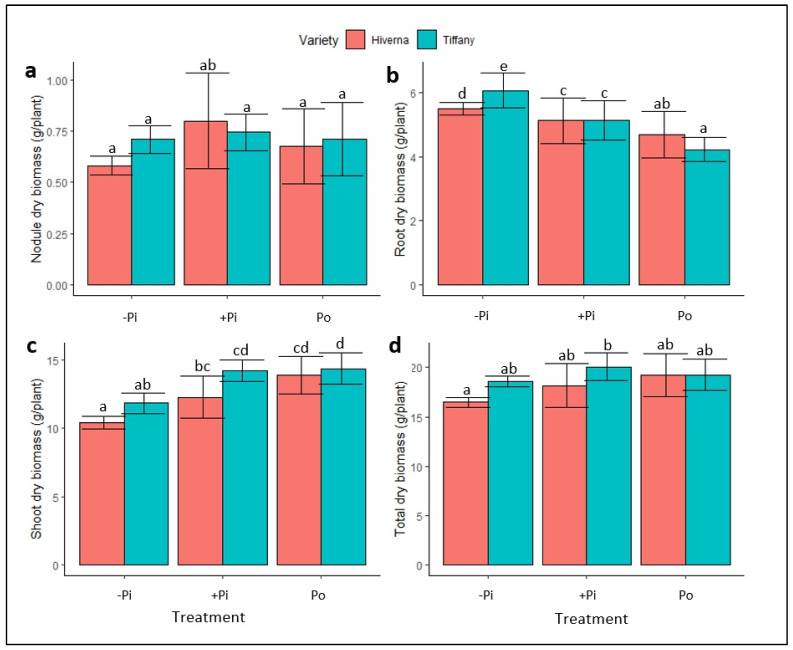
Dry biomass of faba bean (Tiffany and Hiverna) varieties treated with low KH_2_PO_4_ (−Pi), adequate KH_2_PO_4_ (+Pi), and phytic acid (Po), inoculated with *Rhizobium leguminosarum bv. Viciae* 3841, and grown in a hydroponic experiment for 30 days. (**a**) Nodule dry biomass, (**b**) root dry biomass, (**c**) shoot dry biomass, and (**d**) total dry biomass. Data represent the means ± SDs of four biological replicates. Data with different letters are significantly different, as determined by Tukey’s test (*p* ≤ 0.05).

A two-way analysis of variance showed significant effects of P treatments on a shoot dry biomass (SDB), root dry biomass (RDB), and overall dry biomass (TDB), but not on nodule dry biomass (NDB) (Figure 1 and Figure S1c). The results showed no significant differences between the varieties in NDB, but significant differences (*p* ≤ 0.05) were observed in terms of RDB under −Pi (Figure 1a,b). P supplementation showed no effect on NDB; however, RDB under insufficient P recorded the highest root biomass, as expected under such conditions (Figure 1b), with Po-treated varieties recording the least. The Tiffany variety accumulated the greatest RDB under inadequate P treatment (Figure 1b). Meanwhile, Po-treated varieties obtained the greatest SDB, with Tiffany (21 and 1%) recording the highest SDB relative to inadequate and adequate Pi (Figure 1c), respectively. The overall biomass showed no significant variation due to the higher root biomass observed under −Pi, but Tiffany supplied with adequate +Pi was the highest (Figure 1d). The treatment × variety (T × V) interaction was nonsignificant in the measured traits.

**Figure 2:**
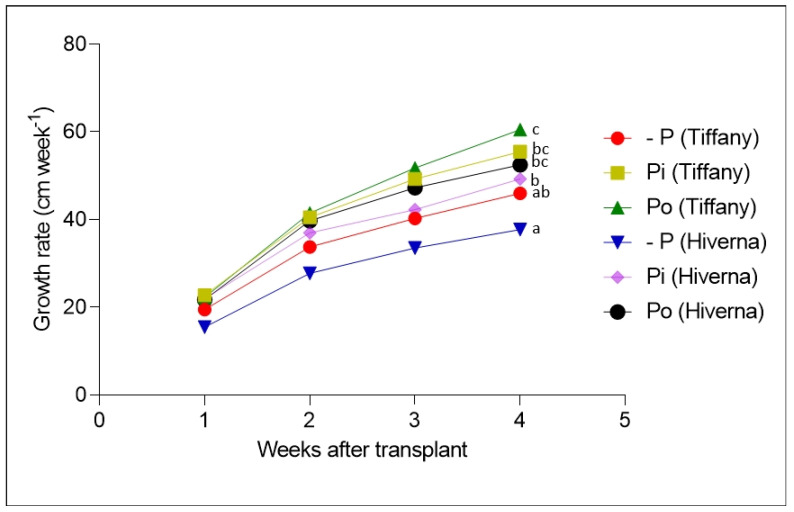
Weekly growth of faba bean (Tiffany and Hiverna) varieties treated with low KH_2_PO_4_ (−Pi), adequate KH_2_PO_4_ (+Pi), and phytic acid (Po), inoculated with *Rhizobium leguminosarum bv. viciae* 3841, and grown in a hydroponic experiment for 30 days. Data represent the means ± SDs of four biological replicates determined by a Tukey’s test (*p* ≤ 0.05). Data with different letters are significantly different, as determined by Tukey’s test (*p* ≤ 0.05).

**Figure 3:**
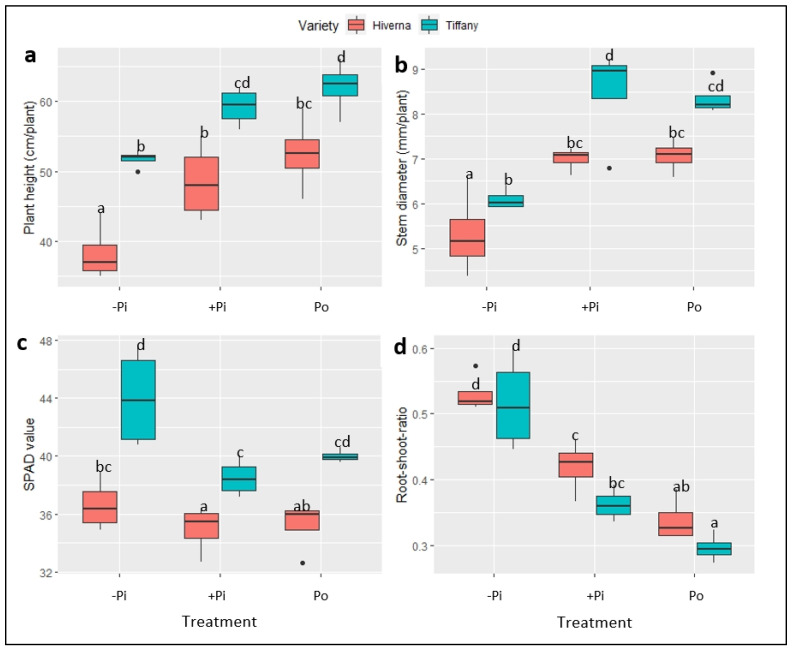
Growth and physiology of faba bean (Tiffany and Hiverna) varieties supplemented with low KH_2_PO_4_ (−Pi), adequate KH_2_PO_4_ (+Pi), and phytic acid (Po), inoculated with *Rhizobium leguminosarum bv. viciae* 3841, and grown in a hydroponic experiment for 30 days. (**a**) Plant height, (**b**) stem diameter, (**c**) chlorophyll content, and (**d**) root-shoot ratio. Data represent the means ± SDs of four biological replicates. Data with different letters are significantly different, as determined by Tukey’s test (*p* ≤ 0.05).

Figure 2 and Figure 3 show the growth of faba bean varieties supplemented with different P. The results on plant height (PH), stem diameter (SD), root/shoot ratio (RSR), and SPAD (soil–plant analytical development) showed significant variation (*p* ≤ 0.01 and 0.001, respectively) among treatments and varieties. Data on PH and SD indicate that the Tiffany variety recorded the highest growth compared with Hiverna under all three treatments (Figure 3a,b). However, no significant differences existed between +Pi and Po, but both differed markedly from their corresponding −Pi-treated varieties. Interestingly, Tiffany under −Pi recorded similar PH and SD as Hiverna under both treatments (+Pi and Po) (Figure 3a,b). In terms of PH and weekly growth, Po-supplemented Tiffany and Hiverna increased by 5% and 8%, respectively, compared to +Pi-fed varieties (Figure 3a,b). The growth of Tiffany followed a similar pattern as the PH (Figure 2 and Figure S1a,b). However, P application did not significantly affect SPAD, with Tiffany under −Pi recording the highest chlorophyll content in this regard (Figure 3c). Generally, Tiffany plants observed the greatest SPAD value under all three treatments compared with Hiverna. Accordingly, no significant differences were observed between both treatments (+Pi and Po). The RSR is measured to characterize the effect of P stress on below-ground biomass, and observing a higher RSR under −Pi in this work was highly anticipated (Figure 3d). Statistically significant treatment differences (*p* ≤ 0.001) were observed among treatments, with Po recording the lowest RSR. However, the Hiverna variety obtained the highest RSR compared with Tiffany in all treatments. Meanwhile, T × V interactions were not informative in all measured traits, as depicted (Figure 2 and Figure 3).

### 2.2. Phosphorus and Other Elemental Nutrient Concentrations in Tissues

**Figure 4:**
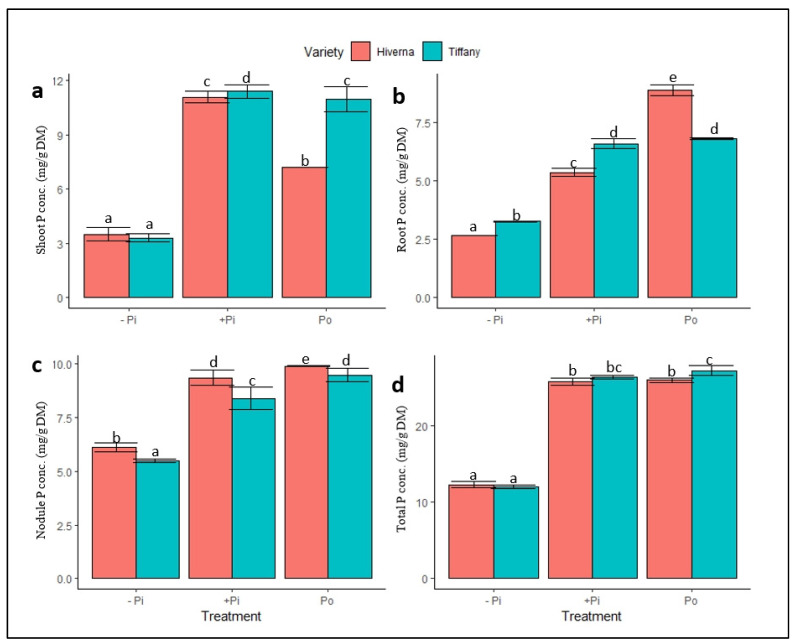
Normalized P levels in tissues of Tiffany and Hiverna varieties of faba bean supplemented with low KH_2_PO_4_ (−Pi), adequate KH_2_PO_4_ (+Pi), and phytic acid (Po), inoculated with *Rhizobium leguminosarum bv. viciae* 3841, and grown in a hydroponic experiment for 30 days. (**a**) Shoot P, (**b**) root P, (**c**) nodule P, and (**d**) total P. Data represent the means ± SDs of four biological replicates. Data with different letters are significantly different, as determined by a Tukey’s test (*p* ≤ 0.05).

**Figure 5:**
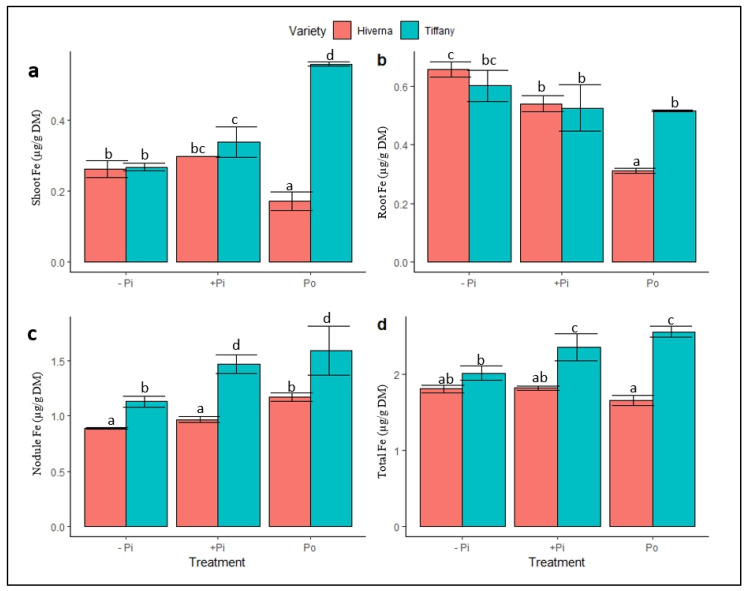
Iron (Fe) concentrations in tissues of Tiffany and Hiverna varieties of faba bean supplemented with low KH_2_PO_4_ (−Pi), adequate KH_2_PO_4_ (+Pi), and phytic acid (Po), inoculated with *Rhizobium leguminosarum* bv. *viciae* 3841, and grown in a hydroponic experiment for 30 days. (**a**) Shoot Fe, (**b**) root Fe, (**c**) nodule Fe, and (**d**) total Fe. Data represent the means ± SDs of four biological replicates. Data with different letters are significantly different, as determined by Tukey’s test (*p* ≤ 0.05).

**Figure 6:**
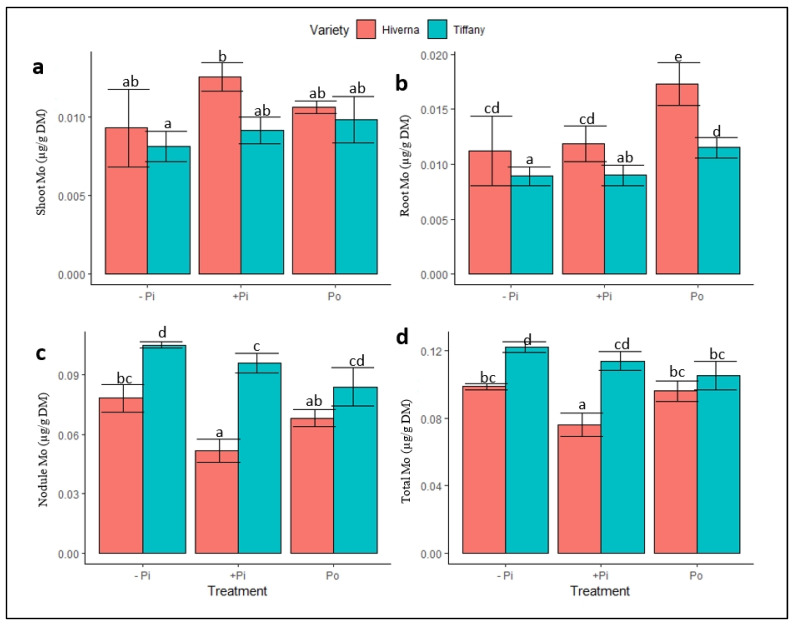
Molybdenum (Mo) concentrations in tissues of Tiffany and Hiverna varieties of faba bean treated with low KH_2_PO_4_ (−Pi), adequate KH_2_PO_4_ (+Pi), and phytic acid (Po), inoculated with *Rhizobium leguminosarum bv. viciae* 3841, and grown in a hydroponic experiment for 30 days. (**a**) Shoot Mo, (**b**) root Mo, (**c**) nodule Mo, and (**d**) total Mo. Data represent the means ± SDs of four biological replicates. Data with different letters are significantly different, as determined by Tukey’s test (*p* ≤ 0.05).

Differences in P availability affected the P status and other mineral nutrients in faba bean nodules, roots, and shoots. Contrasting P, Fe and Mo concentrations in Tiffany and Hiverna were observed under the different P sources and tissues (Figure 4, Figure 5 and Figure 6). The two-way ANOVA analysis of minerals revealed marked differences in the distribution of P, as P concentrations increased upon supply and differed significantly between (*p* ≤ 0.05, 0.01, 0.001) treatments and varieties across all tissues (Figure 4). Shoot P concentration (SP_Conc_) differed significantly (*p* ≤ 0.05) between the varieties under both P treatments, with Tiffany obtaining the greatest in this regard (Figure 4a). Root P concentration (RP_Conc_) also exhibited similar trends to the shoot except under Po treatment, where Hiverna accumulated the highest P (Figure 4b). On the contrary, nodules P concentration (NP_Conc_) showed different trends when the varieties were compared. Hiverna obtained the greatest NP_Conc_ and differed markedly from Tiffany across all treatments (Figure 4c). The NP_Conc_ was comparable to the shoot, but was higher than the root when treatments were summed. The overall elemental P concentration (TP_Conc_) showed significant variation in treatments, but not varieties (Figure 4d). T × V interactions were statistically significant (*p* ≤ 0.05, 0.01, 0.001) in all measured traits in the various treatments and varieties (Figure 4). Interestingly, overall elemental P contents differed significantly between treatments (*p* ≤ 0.001) and varieties (*p* ≤ 0.05) (Table 1). However, +Pi and Po treatments did not vary in their P contents, but changed significantly from the −Pi. The results showed that Tiffany recorded 10% more elemental P content than Hiverna. In Hiverna, Po-treated varieties observed an increase of 6% more elemental P content than +Pi (Table 1).

Fe and Mo were determined because they are the principal components of the nitrogenase complex. This was to assess the effects of Po supply on the uptake and metabolism of both elements in the nodules. The Fe concentration in tissues exhibited a contrasting response upon P supply (Figure 5). The tested ANOVA showed significant differences between them (*p* ≤ 0.05, 0.01, 0.001). The Fe concentration in shoots, nodules, and total Fe were higher in Tiffany compared with Hiverna except in the root (Figure 5d). The overall nodule Fe concentrations under −Pi increased by 61 and 283%, respectively, relative to roots and shoots (Figure 5c). Under adequate +Pi, the nodule Fe increased by 131 and 286%, respectively, compared to roots and shoots (Figure 5c). In comparison to roots and shoots under Po-treated plants, nodule Fe levels elevated by 219 and 263%, respectively. T × V interactions were statistically informative (*p* ≤ 0.05, 0.01, 0.001) in all measured traits in the various treatments and varieties.

Normalized molybdenum (Mo) concentrations in Tiffany and Hiverna tissues portrayed contrasting responses under the different P sources (Figure 6). Significant differences were observed in the treatments (*p* ≤ 0.05, 0.01) and varieties (*p* ≤ 0.05, 0.001). However, Mo concentrations were higher in the shoot and root of the Hiverna variety (Figure 6a,b), but the reverse was observed in the nodule and total Mo, with the Tiffany variety recording the highest (Figure 6c,d). The nodules Mo concentrations were 850 and 1018% higher than roots and shoots under −Pi (Figure 6c). While under +Pi, the nodules Mo increased by 614 and 582%, respectively, relative to their corresponding roots and shoots. Additionally, nodule Mo increased by 424 and 630%, respectively, compared to roots and shoots under Po treatment. Overall, Mo concentration was higher in nodules compared to other tissues. Similarly, T × V interactions were only statistically informative (*p* ≤ 0.05, 0.01, 0.001) in the nodule and overall Mo concentration in the various treatments and varieties. 

The total Mo and Fe concentrations correlated positively with each other (r = 0.39, *p* ≤ 0.05), but Mo concentration correlated negatively with P concentration (r = −0.03, *p* ≤ 0.05). However, Fe content observed a positive correlation with P content (r = 0.3, *p* ≤ 0.05) (Figure S3).

### 2.3. Effect of Po Supply on Inorganic Anions Concentrations in Tissues

**Figure 7:**
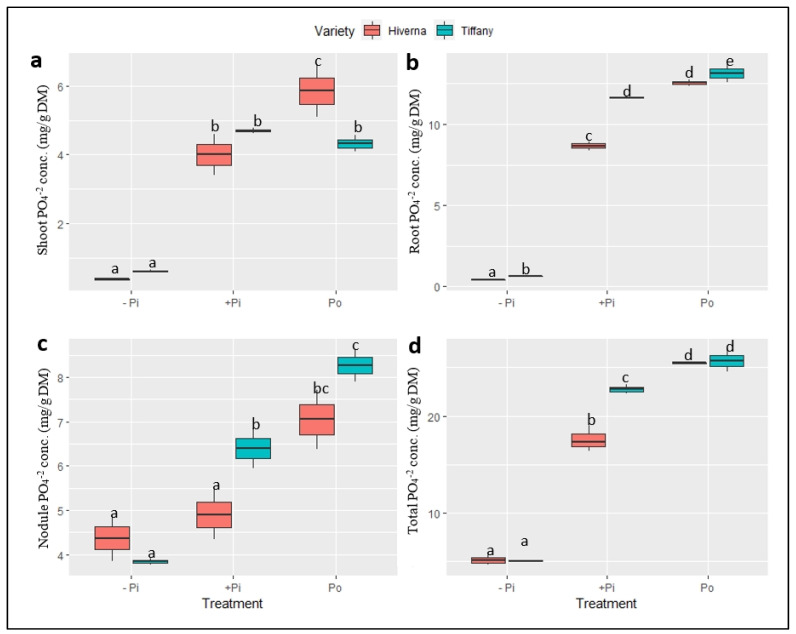
Phosphate (PO_4_^3−^) concentrations in tissues of Tiffany and Hiverna varieties of faba bean supplemented with low KH_2_PO_4_ (−Pi), adequate KH_2_PO_4_ (+Pi), and phytic acid (Po), inoculated with *Rhizobium leguminosarum bv. viciae* 3841, and grown in a hydroponic experiment for 30 days. (**a**) Shoot PO_4_^3−^, (**b**) root PO_4_^3−^, (**c**) nodule PO_4_^3−^, and (**d**) total PO_4_^3−^. Data represent the means ± SDs of four biological replicates. Data with different letters are significantly different, as determined by a Tukey’s test (*p* ≤ 0.05).

**Figure 8:**
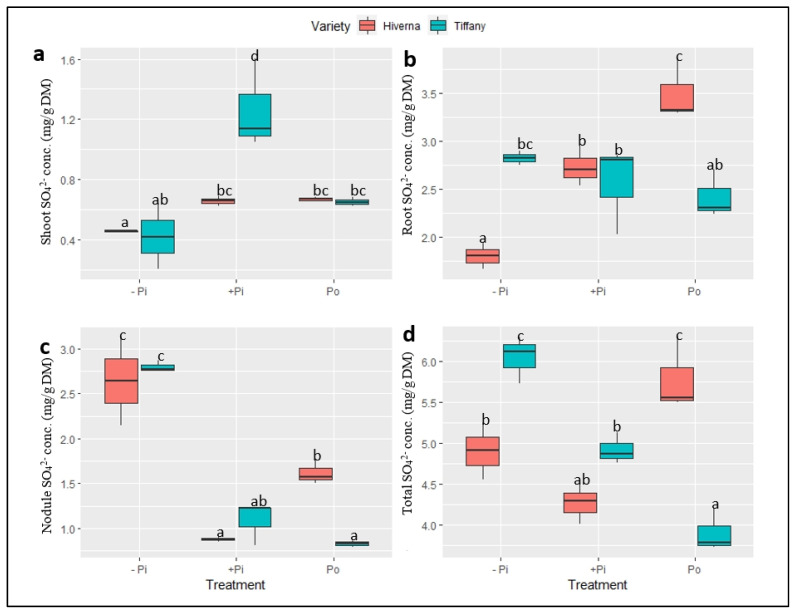
Sulphate (SO_4_^2−^) concentrations in tissues of Tiffany and Hiverna varieties of faba bean supplemented with low KH_2_PO_4_ (−Pi), adequateKH_2_PO_4_ (+Pi), and phytic acid (Po), inoculated with *Rhizobium leguminosarum bv. viciae* 3841, and grown in a hydroponic experiment for 30 days. (**a**) Shoot SO_4_^2−^, (**b**) root SO_4_^2−^, (**c**) nodule SO_4_^2−^, and (**d**) total SO_4_^2−^. Data represent the means ± SDs of four biological replicates. Data with different letters are significantly different, as determined by a Tukey’s test (*p* ≤ 0.05).

The analysis of various anions revealed marked differences in the accumulation and distribution of PO_4_^3−^ and SO_4_^2−^ in nodules and other tissues grown under different P fertilization sources (Figure 7 and Figure 8). The results showed a marked increase in PO_4_^3−^ levels upon P supply across tissues (Figure 7). Highly significant differences (*p* ≤ 0.001) were observed across measured tissues in both treatments and varieties. Shoot phosphate concentration was generally low, but higher in root and nodules (Figure 7a–c). Compared to other treatments, Po was comparatively the best in all tissues and differed significantly (*p* ≤ 0.001) from +Pi. The overall PO_4_^3−^ levels accumulated by Tiffany were higher compared with Hiverna (Figure 7d). Additionally, T × V interactions were highly informative (*p* ≤ 0.001) in all tissues and overall concentrations in the various treatments and varieties.

The normalized sulphate (SO_4_^2−^) concentrations in tissues of Tiffany and Hiverna varieties of faba bean supplemented with different P sources also showed highly significant variations in the treatments (*p* ≤ 0.001), but generally not in the varieties (Figure 8). Overall, SO_4_^2−^ concentrations were higher in roots, with the shoot recording the least (Figure 8a,b). However, nodule SO_4_^2−^ concentrations under −Pi in both varieties were at least 1.5-fold higher than both +Pi and Po treatments (Figure 8c). Statistically significant T × V interactions were observed (*p* ≤ 0.001) in all tissues and the overall concentration.

### 2.4. Carbon Metabolite Concentrations in Tissues in Response to Phytic Acid Supply 

**Figure 9:**
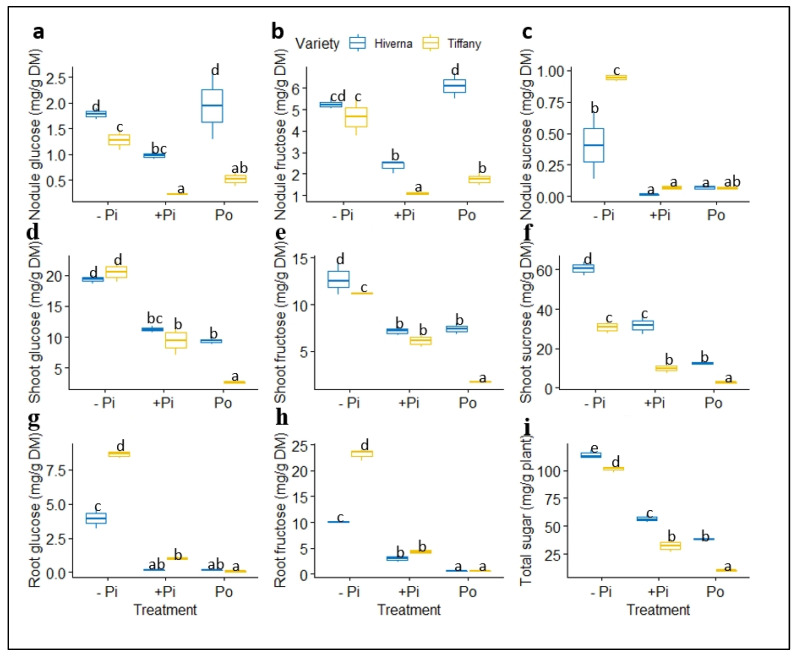
Soluble sugar concentrations in tissues of Tiffany and Hiverna varieties of faba bean fertilized with low KH_2_PO_4_ (−Pi), adequate KH_2_PO_4_ (+Pi), and phytic acid (Po), inoculated with *Rhizobium leguminosarum bv. viciae* 3841, and grown in a hydroponic experiment for 30 days. (**a**) Nodule glucose, (**b**) nodule fructose, (**c**) nodule sucrose, (**d**) shoot glucose, (**e**) shoot fructose, (**f**) shoot sucrose, (**g**) root glucose, (**h**) root fructose, and (**i**) total sugar. Data represent the means ± SDs of four biological replicates. Data with different letters are significantly different, as determined by a Tukey’s test (*p* ≤ 0.05).

**Figure 10:**
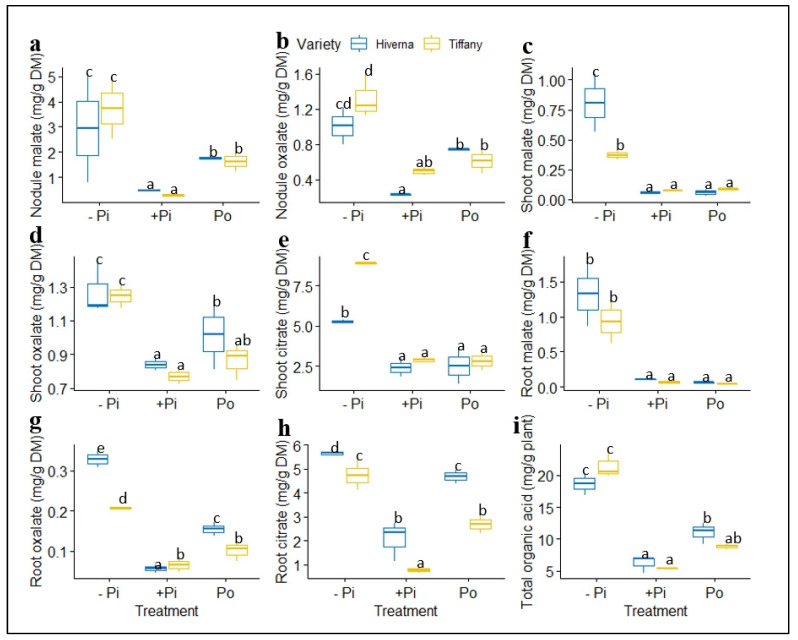
Normalized organic acids concentrations in tissues of Tiffany and Hiverna varieties of faba bean fertilized with low KH_2_PO_4_ (−Pi), adequate KH_2_PO_4_ (+Pi), and phytic acid (Po), inoculated with *Rhizobium leguminosarum* bv. *viciae* 3841, and grown in a hydroponic experiment for 30 days. (**a**) Nodule malate, (**b**) nodule oxalate, (**c**) shoot malate, (**d**) shoot oxalate, (**e**) shoot citrate, (**f**) root malate, (**g**) root oxalate, (**h**) root citrate, and (**i**) total organic acid. Data represent the means ± SDs of four biological replicates. Data with different letters are significantly different, as determined by a Tukey’s test (*p* ≤ 0.05).

Identified metabolites showed great significant differences (*p* ≤ 0.001) in both treatments and varieties (Figure 9 and Figure 10). Low Pi supply increased the level of identified sugars (glucose, fructose, and sucrose) in nodules. However, the Hiverna variety supplied with Po also accumulated higher glucose and fructose, as in −Pi (Figure 9a–c). Comparatively, nodule sucrose concentration was generally lower than glucose and fructose in nodules and other sugars in roots and shoots (Figure 9c). Shoot sugar concentration increased tremendously under −Pi supply and differed significantly (*p* ≤ 0.001) among the different treatments (Figure 9d–f). Significant differences were comparable on the individual variety level except in Tiffany plants supplied with Po, where some significant level was observed between the two varieties. Shoot sucrose was observed to be several folds higher than glucose and fructose (Figure 9d–f).

Contrary to shoots where metabolite levels were comparable among the two P treatments (+Pi vs. Po), in roots, highly significant variations were observed in the identified sugars, especially in fructose (Figure 9g–h). Additionally, highly significant differences were observed between the two varieties, with Tiffany recording the greatest glucose and fructose. However, the overall sugar concentrations in the entire plant indicated that the Hiverna variety accounted for the greatest sugars compared with Tiffany (Figure 9i). Interestingly, +Pi-treated plants accumulated more sugars than Po-fertilized plants. Sucrose levels were negligible in the roots of the faba bean and so could not be detected. T × V interactions in metabolites were highly significant (*p* ≤ 0.001) in all tissues and the overall concentration.

The total levels of detected organic acids (OAs) (malate and oxalate) in nodules of the two varieties of faba bean were similar under −Pi (Figure 10a,b). P-deficient conditions caused an increase in malate and oxalate and differed significantly from other treatments. Nevertheless, Po-treated varieties increased tremendously compared with the corresponding +Pi in nodules (Figure 10a,b). Citrate concentrations were very negligible and therefore could not be detected in nodules under the three P treatments. However, several individual OAs showed a remarkable change in their level in shoots (Figure 10c–e). Shoot malate and citrate exhibited highly significant differences between (*p* ≤ 0.001) Tiffany and Hiverna under −Pi condition, with Hiverna and Tiffany recording the greatest in malate and citrate, respectively (Figure 10c–e). OAs concentration in shoots was comparable and similar among the two sufficient P treatments. Contrary to what was observed in shoots, root malate under inadequate P was similar for both varieties, but differed among treatments (Figure 10f). However, both varieties and treatments differed significantly in oxalate and citrate, with Hiverna accumulating the highest under −Pi conditions.

**Figure 11:**
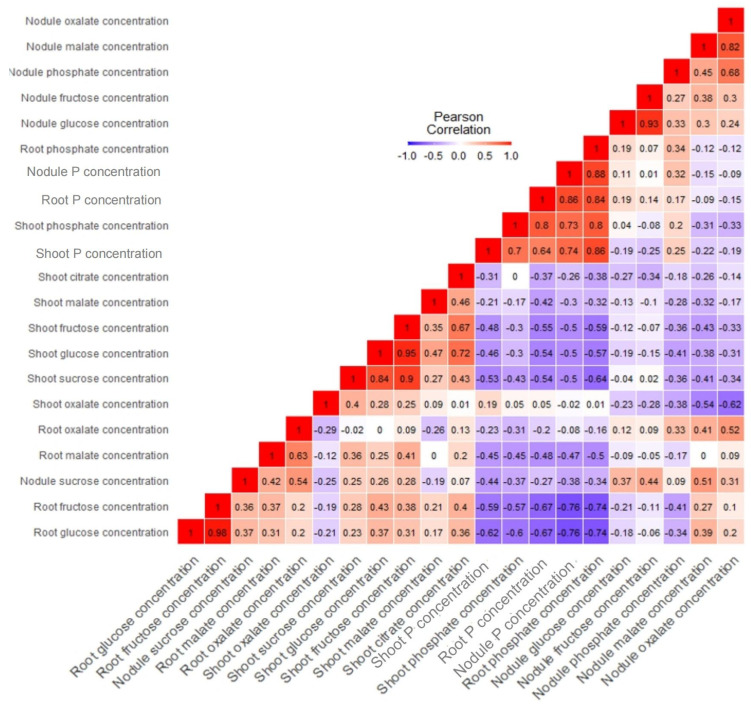
Heat map of correlations of tissue-specific metabolites, anions, and P level traits of Tiffany and Hiverna varieties fertilized with low KH_2_PO_4_ (−Pi), adequate KH_2_PO_4_ (+Pi), and phytic acid (Po), inoculated with *Rhizobium leguminosarum bv. viciae* 3841 and grown in a hydroponic experiment for 30 days. Created following Pearson’s r_s_ values. Color, values, and squares denote Pearson’s rank correlation r between traits, with four biological replicates.

Meanwhile, root oxalate and citrate were greater in Po than with +Pi (Figure 10g,h). The overall OAs concentrations in the plant indicated that Po was 70% higher than the +Pi treatment (Figure 10i). The tested ANOVA showed that T × V interaction was highly significant across all tissues. The data were subjected to correlations to ascertain the relationships among metabolites and P concentrations in all tissues (Figure 11). The results showed strong negative and significant correlations between metabolites and P accumulation. Root, shoot, and nodule P and phosphate concentrations correlated negatively with almost all sugars and OAs. For instance, root glucose correlated negatively with root phosphate concentration (r = −0.74, *p* ≤ 0.05). However, shoot oxalate observed a positive, but nonsignificant correlation with shoot P (r = 0.19, *p* ≤ 0.05). Nodule sucrose correlated positively and significantly with nodule malate (r = 0.51, *p* ≤ 0.05, Figure 11).

**Figure 12:**
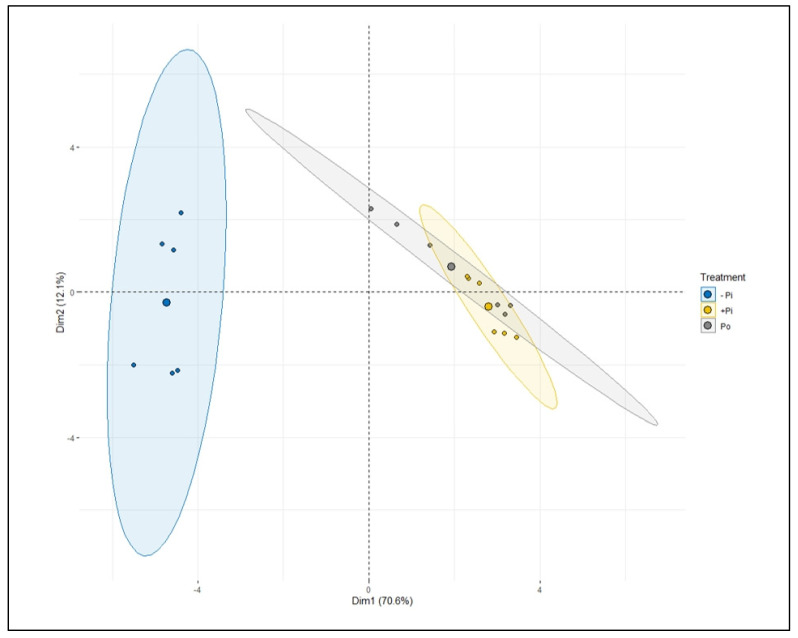
Principal component analysis (PCA) showing the relationship between treatments’ metabolic changes (i.e., sugars and organic acids) in Tiffany and Hiverna in response to low KH_2_PO_4_ (−Pi), adequate KH_2_PO_4_ (+Pi), and phytic acid (Po), inoculated with *Rhizobium leguminosarum bv. viciae* 3841, and grown in a hydroponic experiment for 30 days.

A principal component analysis (PCA) was conducted as an alternative statistical approach to appraise and establish the correct variations in the various metabolites and other measured traits. This method distinguished a highly significant variation across treatments and traits (Figure 12 and Figure S2). The first two principal components (PC) explained 70.6 and 12.1% of the variation, respectively, in the two varieties (Figure 12). The other PC explained 31 and 16.1% of the variation (Figure S2). ANOVA was carried out on each PC to evaluate if treatments differed among varieties and traits. The results, however, indicated highly significant differences between sufficient (Pi and Po) and insufficient P plants, as illustrated (Figure S2). PC1 and PC2 largely separated -P treatments from P treatments (Pi and Po), indicating Pi and Po are closely related and nonsignificant (Figure 12).

### 2.5. Dynamics of N Content and Fixation in Tissues

The mean total N concentration for the whole plant of faba bean varieties was 11.7%, with significant differences (*p* ≤ 0.01) between the treatments, but not the varieties (Table 1). The highest N concentration was recorded in nodules ranging from 6.0% to 5.4%, with no significant differences between the treatments. No significant differences were observed between the varieties and treatments for the root and shoot N concentration (Table 1). 

The N content observed significant differences in the shoot, root, and overall content, except in the nodules (*p* ≤ 0.05) of both treatments and varieties (Table 1). However, both P treatments (+Pi vs. Po) were nonsignificant in the total N content. The contents of N in the shoot were 2–10-fold higher than those of the root and nodule, respectively. The entire N content indicated that Tiffany was 10% higher than Hiverna, and T × V interactions were nonsignificant across tissues and the overall N content. 

The δ^15^N of shoots, roots, and nodules was not significantly different between the treatments and varieties (Table 1). All the treatments and varieties exhibited negative δ^15^N values for all tissues in the range of −2.0‰ to −3.7‰. No significant differences were observed for the corrected tissue δ^15^N values. However, roots observed lower δ^15^N compared to the other tissues. 

The percentage of symbiotic N_2_ fixation by faba bean (%N_dfa_) showed significant differences in both treatments and varieties and ranged from −46.7 to −130.3% in the shoot, with all negative values (Table 1). However, root %N_dfa_ was generally low, with both positive (in Hiverna) and negative (in Tiffany) values and significant differences between the treatments and varieties. Root %N_dfa_ ranged from −7.0 to 8.8% and 2.73 to 15.3% for Tiffany and Hiverna. When the two P treatments were compared, highly significant differences were observed, with Hiverna recording more positive values (Table 1). 

Phosphorus/nitrogen ratio (P/N ratio) observed highly significant differences (*p* ≤ 0.001) between treatments, but not in the different varieties, with −Pi being the most affected treatment (Table 1). Comparatively, Po-supplemented plants differed significantly from +Pi in both varieties. For instance, the results showed that Po increased by 12% and 24% in Tiffany and Hiverna, respectively, relative to their respective +Pi-treated plants in their P/N ratio. However, the results on the P/N ratio showed that the Tiffany variety observed an increase of 2% more than Hiverna, with no significant interaction between treatments and varieties (Table 1).

## 3. Discussion

### 3.1. Phytic Acid Supply Improves Growth and Partitions Higher Biomass, Similar to Inorganic P

Prior to this work was an initial soil experiment aimed at assessing the effects of Po supply on the growth and efficient utilization of Po by diverse varieties of faba bean [22]. By changing the growth medium (from soil to hydroponics), we have confirmed and concluded that faba bean plants are competent users of phytic acid and accumulate more total biomass, which is better or equivalent to Pi-supplemented faba bean plants. This finding supports an earlier premise that Po could serve as a perfect alternative to Pi [17]. 

This paper thus presents an attestation that faba bean varieties can utilize Po, with Po supply imposing a staunch footprint on the growth and development of plants when exclusively depending on N derived from symbiotic nitrogen fixation, not only in soils [22], but also in hydroponic systems. The results showed significant differences in traits such as SDB, RDB, PH, and SD between treatments (Figure 1, Figure 2 and Figure 3). This response was underpinned by a significant increase in RDB, which assisted plants in obtaining Pi from phytate substrates [17]. However, P supplementation did not impact NDB or SPAD (Figure 1, Figure 2 and Figure 3). Previous evidence has suggested that plants supplied with Po can amass more or equivalent biomass to Pi [14,16,21,22]. In this present study, Po treatment obtained the highest SDB (3%), PH, and growth rate (5 and 8%) in Tiffany and Hiverna, respectively, compared with +Pi. Adams and Pate [14] observed similar results in two lupin species (*L. albus* and *L. angustifolius*), with dry matter increasing greatly when inositol-P was supplied, supporting the hypothesis that Po could be a game-changer in P nutrition in the foreseeable future [17]. Additionally, Li et al. [20] observed similar biomass in chickpea plants supplied with phytate and KH_2_PO_4_ in monoculture and intercropped in a hydroponics system. They observed no significant differences in both organic and inorganic P treatments. Accordingly, the Tiffany variety observed higher overall biomass than Hiverna, confirming the earlier assertion and its competent Po utilization efficiency [22]. 

Root characteristics (e.g., biomass and RSR) are suggested to play an adaptive role during P limitation. Plants with higher root biomass under stress have been observed to exhibit better P scavenging ability and absorption rates [23,24]. Considerable studies have reported that plants under deficient P conditions accumulate higher root biomass by allocating most of their photosynthates to the below-ground organs (root) to acclimatize to the deleterious P effects [25,26]. A similar trend was observed in this present study, where under the −Pi condition, faba bean plants recorded the highest RDB and RSR (Figure 1, Figure 2 and Figure 3), resulting in the nonsignificant differences between treatments in the overall biomass. Apparently, RSR is measured to characterize the effect of P stress on below-ground biomass; therefore, observing higher RSR under −Pi in this work supports the above premise. It is well documented that P deficiency significantly impacts nodule growth and reduces N_2_ fixation efficiency [9,10,27]. Unfortunately, this was not the case in this study, as P application did not influence nodule biomass. The nonsignificant differences between treatments in nodules could be attributed to the fact that nodules continued to grow even when P supply to the growing plant was very limited, since P is always optimum in nodules.

### 3.2. Phytic Acid Supply Alters Nutritional Distribution and Allocation in the Nodules 

The significant role that mineral nutrient [6] and other inorganic anions [28,29] play in the growth and cellular functions of microbes and their synergistic interactions in legumes have been reported. The absence of minerals such as P, Fe, Mo, and S has been shown to have a detrimental effect on symbiotic nitrogen fixation (SNF) in legumes [6,30]. 

The analysis of the results consistently revealed an increase in Pi and PO_4_^3−^ in nodules similar to the shoot and root upon P supply (Figure 4 and Figure 7). Likewise, under inadequate P conditions, the nodule P was substantially higher than the other tissues. Substantial evidence has indicated that nodules are preferential Pi sink organs, which accumulate relatively higher Pi concentrations than other plant organs [31,32]. This phenomenon is probable because nodule functioning requires excessive carbon, ATP, and NADH [33,34]. Indeed, the higher P observed in nodules compared to other organs supports the premise that nodules are preferential organs and require higher amounts of P for proper Pi homeostasis [7,9,10,27,35]. Therefore, it is quite tempting to conclude that Tiffany under Po treatment has a propensity for higher internal Pi remobilization and reallocation to nodules for proper functioning [27]. Meanwhile, the overall P concentration did not differ at both treatments and varietal levels (Figure 4d), suggesting that the P-treated (+Pi vs. Po) plants acquired P in a comparable concentration, as previously reported [16]. This phenomenon was observed by Li et al. [20], when chickpea plants treated with phytate in hydroponics observed higher P concentrations than KH_2_PO_4,_ but differed significantly in maize under similar conditions. This study hypothesized that the higher P content induced under Po could play a significant role in nodule functioning and reprogramming. Interestingly, the Po-treated plants did not only accumulate the highest Pi and PO_4_^3−^ concentrations in nodules, but also obtained the greatest in roots and shoots relative to +Pi (Figure 4 and Figure 7). The high accumulation of P content under Po supply not only signifies that *Vicia faba* plants have a strong capacity to utilize Po substrates, but also have the ability to drastically lessen the energetic costs when plants are exclusively relying on nodule symbiosis as a sole source for N. 

The formation of the nitrogenase complex demands not only optimum amount of Fe and Mo levels for the synthesis of the oxygen-sensitive Fe- and Mo-Fe complexes [36,37], but also requires sufficient sulfate (S), which is a principal component for the cellular synthesis of the metal S-centers of the nitrogenase complex of SNF in legumes [6,36]. These elements are critical for N_2_-fixing legumes [38,39], with Fe being the dominant component of the leghemoglobin protein, which regulates oxygen exchange in the bacteroids [39]. Our results indicated that substantial amounts of Fe, Mo, and S were accumulated in nodules relative to other organs under the same application rates (Figure 5, Figure 6 and Figure 8). Indeed, the observed increases in the accumulation of these nutrients exemplify that these three nutrients are critical components of the nitrogenase complex. The simple explanation is that these elements are needed in large quantities to synthesize the nitrogenase complex (enzymes) that executes the conversion of atmospheric N_2_ to ammonia for assimilation by plants. The result agrees with those of [6], where Fe, Mo, and S were higher in infected cells and the bacteria-infected nodules in *Psoralea pinnata* plants grown in wetland and upland conditions. Although the results were contrasting among the varieties, the total Fe and Mo concentrations generally indicated that the Tiffany variety was the best, with better reallocation capacity than Hiverna. The differences were due to their differential mechanisms, genetics, and differential acquisition efficiencies [22]. Interestingly, the results consistently revealed that faba bean plants supplied with phytic acid improved the overall Fe and Mo concentrations in Tiffany varieties compared with Hiverna (Figure 5d and Figure 6d). This suggests that remobilization and utilization of Fe and Mo are highly enhanced and modulated by Po supply and are species specific. 

Furthermore, the results highlight that Po plays crucial and synergistic roles in the uptake of Fe and Mo in Tiffany during SNF. Interestingly, S concentration in nodules under −Pi was remarkably higher than the corresponding sufficient P treatments, although S was supplied in equal amounts. This increase in S under low P supply relative to high P reflects the higher S demand for the functioning nodules and that nodules could maintain their SNF capacity even under limited P conditions by preferentially reallocating the S to the nodules. The higher concentration of S under poor S conditions has also been reported [40]. Even though the S accumulation in both varieties and treatments was apparently contrasting, we can conclude that the singular effect of Po supply on internal S distribution and allocation was effective relative to +Pi. Hence, it is quite plausible to suggest that the higher Po content did not only improve nitrogenase-synthesizing nutrients, but also contributed significantly to growth and Pi homeostasis in the functioning nodules.

### 3.3. Phytic Acid Supply Furnishes the Growing Nodules with Higher Carbon (Malate) for Nodule Functioning 

Biomass partitioning and other biochemical mechanisms have been reported to be involved in organic P acquisition and utilization by *Vicia faba* plants [21,22]. Established shreds of evidence indicate that the exudation of organic acids (OAs) such as malate and citrate is predominantly required for adaptation during P starvation in plants [41,42,43]. The exudation of higher amounts of OAs that sanction the chelation and sequestration of Al^3+^, Fe^3+^, and Ca^2+^, and the subsequent hydrolysis and release of fixed P from precipitated forms have been chronicled [5,44,45]. Notwithstanding, the higher exudation and accumulation of these organic substances can have deleterious effects by draining the carbon (C) budget, thereby lessening the availability of C for other processes in the plant [21,46]. 

This present study showed that the release of overall OAs (malate, oxalate, and citrate) from the nodules, roots, and shoots of −Pi-supplemented faba bean plants increased 243 and 102% more than those plants receiving +Pi and Po, respectively (Figure 10i). This is more likely to cause rhizodeposition, as previously suggested [21], and is also known to be used as an adaptation strategy [41,43,44] and a recurrent characteristic of P deficiency in plants [11,47,48]. The marked increase in OAs under P starvation in this study agrees with what has been reported under symbiotic conditions in response to P treatments [5,12,38]. Similarly, the nodules of plants induced by phytic acid significantly increased malate and oxalate concentrations compared with +Pi (Figure 10a,b). Interestingly, the overall OAs between the two P treatments (+Pi vs. Po) indicated that Po-treated plants increased by 70% more than +Pi (Figure 10i). As reported earlier, nodules sometimes act similarly to roots and exude OAs that enhance nodule Pi homeostasis [21,27]. The reported findings of our study agree with Jillani et al. [21], where the application of phytic acid induced higher OAs compared with KH_2_PO_4_. This observation could suggest that Po-treated plants exuded more OAs than +Pi, and hence, proposed to improve nodulation and the SNF and P nutrition of plants. Therefore, the substantial increase in the overall P uptake and content of PO_4_^3−^ in Po-treated plants relative to +Pi is a direct consequence of the concomitant higher OAs exudation under Po treatment. This increase improved the solubilization and efficient uptake of Po substrates by *Vicia faba* plants. 

Malate is a predominant OA in nodules. In fact, it is the primary carbon (C) source that can be utilized by the bacteroids to assist in SNF [49,50]. This indicates that malate must be optimal under certain circumstances to provide the C skeleton for SNF [50]. So, observing higher malate accumulation in nodules relative to other tissues supports the above premise (Figure 10a). Conversely, the higher accumulation of OAs under Po supply compared with sugars in this study implies that nodulating *Vicia faba* plants resorted to an alternative carbon metabolism pathway (i.e., OAs) for C instead of sugars or hexose as depicted (Figure 9). This alternative route occurred because plants that ultimately rely exclusively on nitrogen sourced from nodule symbiosis require higher OAs (malate) to be able to supply the required C for nodule growth and symbiosis. Indeed, it has been reported that nodules cannot directly utilize C from sucrose, but utilize a C source synthesized through the glycolytic pathway to the TCA cycle to make low-molecular-weight organic anions (e.g., malate) available for the nodule [49,50]. Meanwhile, the differential accumulation and exudation of OAs were observed under Po in our results. The results indicated that the Hiverna variety accumulated the highest OAs in both Po- and +Pi-treated plants relative to Tiffany (Figure 10i), suggesting that Hiverna possesses the ability to increase its internal C and Pi metabolism for reallocation to nodules better than +Pi. Put together, the hypothesis that the higher P contents induced by phytic acid supplementation might supply the needed amounts of C to the functioning nodules to improve the C metabolism and mineral contents of the functioning nodules is, however, supported.

### 3.4. N_2_ Fixation Efficiency in Faba Beans in Response to Phytic Acid Supply

Many researchers have reported that the roots of most plant species have positive δ^15^N values, with nodules of the majority of these species always displaying strongly positive δ^15^N signatures [51,52,53]. However, these signatures depend on determinants such as the type of variety, growing conditions, age, and part of the plant sampled for analysis [54]. This study only observed strongly negative nodule, root, and shoot δ^15^N values in both faba bean varieties at the different P treatments, with no significant differences (Table 1). We observed negative δ^15^N signatures for nodules in all four biological replicates of both faba bean varieties. This agrees with Rose et al. [55], who observed all negative values in nodules for field pea and faba bean cultivars. Similar negative nodule δ^15^N values for faba bean (cv. Alameda) were reported [56] at the flowering and maturity stages. However, the findings are at odds with those of Nebiyu et al. [54], where nodules from all six faba bean cultivars examined exhibited positive δ^15^N signatures at the pod filling stage of growth. The δ^15^N values were negative for roots and shoots in this present study. The ^15^N enrichment of root and shoot agrees with those reported [54,56] and partially agrees with Rose et al. [55]. The strongly negative values for nodules and other tissues could be attributed to non-uniform isotopic discrimination against heavier isotopes during atmospheric N_2_ fixation, metabolism, N translocation, and distribution in the plant system in response to P supply. 

The percentage of N derived from the atmosphere (%Ndfa) and biological nitrogen fixation (BNF) for faba beans have received contrasting and inconsistent values across many studies [54]. For instance, Köpke and Nemecek [57] reported a %Ndfa of 96% and Unkovich and Pate [52] had a %Ndfa of 20–97% for faba bean. These inconsistencies sometimes lead to underestimation and overestimation (incongruous values) in many cases. For example, Peoples et al. [58] recorded over 100% for %Ndfa using B values evaluated from Australian soybean and mung bean varieties. They associated these overestimations with different species and geographical locations. The %Ndfa ranged from 46 to 130% in shoots of faba bean varieties in this study (Table 1). However, faba bean varieties supplemented with Po obtained 106% and 130% %Ndfa values for Tiffany and Hiverna, respectively, relative to the +Pi treatments. This incongruous estimation could arise from the differential P uptake and the synergistic effects of Po on N assimilation and distribution in plants. The result still requires further research to ascertain the B values of plants in response to organic P fertilizers.

## 4. Materials and Methods

### 4.1. Biological Materials, Cultivation, and P Treatments

*Vicia faba* L. (var. Tiffany and Hiverna) were selected based on their organic phosphorus use efficiency (PoUE) from our previous experiment in soil [22]. In that experiment, Tiffany was adjudged the best variety, with Hiverna being an intermediate, but was selected for this experiment due to it high nodulation ability. The two varieties were cultivated in hydroponics culture condition in a controlled climatic chamber with a 14/10 h day/night cycle, approximately 20/15 °C day/night temperature, 60% relative humidity, and 300 μmol m^−2^ s^−1^ photosynthetic active radiation for four weeks. Firstly, seeds were surface sterilized with NaClO_4_ solution (5–7% *v*/*v*) for 3 min and then washed thoroughly with deionized water to eliminate all inherent microbes. The seeds were soaked in aerated 1 mM CaSO_4_ solution for 48 h (20 °C) and germinated using the sandwich method for 7–10 days (d), with 3 d in darkness and exposure to light afterwards. Three uniform-sized seedlings were transferred to 5 L plastic pots containing a quarter nutrient solution strength [59] with modifications. The basal nutrient solutions contained 0.5 mM CaCl_2_; 0.5 mM KCl; 0.5 mM MgSO_4_·7H_2_O; 1 mM K_2_SO_4_; 0.5 mM NH_4_NO_3_; 15 μM Fe (III) EDTA; 10 μM H_3_BO_3_; 0.25 μM MnSO_4_·H_2_O; 0.25 μM ZnSO_4_·7H_2_O; 0.22 μM CuSO_4_·5H_2_O; 0.025 μM Na_2_MoO_4_·2H_2_O; 0.25 μM CoCl_2_·6H_2_O; and 0.005 μM NiSO_4_·6H_2_O. The nutrient solution concentration was steadily increased in the order of ½, ¾, and full-strength on the second, third, and fourth day, respectively, to avoid any osmotic stress. The nutrient solution was renewed every 3 d to freshen the exhausted nutrients. Three P treatments were applied as inadequate and adequate, and the sources include low Pi (2.0 µM KH_2_PO_4_; −Pi), adequate Pi (200 µM KH_2_PO_4_; +Pi), and Po (200 µM phytic acid; Po). To avoid initial N deficiency during nodule development, the plants were supplemented with a small amount of N (20 µM N-NH_4_NO_3_) for a period of 10 d. Two weeks after transplant, seedlings were inoculated with *Rhizobium leguminosarum* bv. *viciae* 3841 broth, prepared in accordance with [12]. The experiment was arranged in a completely randomized design with four biological replicates for each treatment, and plants were grown for 30 d. The experiment was repeated not only to ascertain the accuracy of the first results in the first hydroponics experiment, but to induce more nodules for analysis.

### 4.2. Monitoring of Growth and Harvesting

Before harvesting, non-destructive data such as growth (rate), plant height, stem diameter, and SPAD (soil–plant analytical development) were taken. First, all three plants in each pot were measured at weekly (W_1_–W_4_) intervals with a measuring tape (ruler) for 4 weeks. Measurements of stem diameter and chlorophyll content were measured once with a digital veneer caliper (mm) (CD-20CPX, Mitutoya Co. Ltd., Kawasaki, Japan) and Chlorophyll Meter SPAD-502Plus (Konica Minolta Inc., Osaka, Japan), respectively. In addition to the weekly growth measurement, a maximum plant height (cm) was determined from the plastic surface to the tip of the plant with a measuring tape a day before harvesting. Plant organs were fractioned into the nodule, root, and shoot during harvesting. We determined the fresh biomass of the shoots immediately after harvesting and oven-dried until constant weight and dry matter were measured. A fresh root containing nodules of each plant was placed in liquid nitrogen (−90 °C) and stored at −20 °C until the nodules were removed, weighed, and dried at 65 °C for 72 h to determine the dry matter (DM). Other samples of nodules, leaves, and roots from each plant were stored at −20 °C for analytical determinations.

### 4.3. Mineral Analyses of Plant Tissues

Oven-dry plant materials were milled to a fine powder and were used to determine minerals in tissues following Sagervanshi et al. [60]. A 200 mg of the finely ground subsamples were digested with 10 mL of 69% HNO_3_ at 190 °C for 45 min in a microwave oven (MARS 6, Xpress, CEM, Matthews, MC, USA). Subsequently, the digested samples were diluted with Milli-Q water (18.2 MΩ cm conductivity) to 100 mL and stored at 4 °C until analysis. Concentrations of P, iron (Fe), and molybdenum (Mo) were measured by inductively coupled plasma mass spectroscopy (ICP-MS; Agilent 7700, Agilent Technologies Inc.) as described in [61]. N concentration was determined using ~1 mg of the finely ground sample in a Thermo Delta V Plus isotope ratio mass spectrometer (Thermo Scientific, Bremen, Germany) following combustion on a Thermo Flash EA 1112 elemental analyzer (Thermo Scientific, Bremen, Germany). The ^15^N natural abundance was determined following [56], while the N derived from the atmosphere (%Ndfa) was quantified and expressed as a percentage using the standard equation proposed in [62]. The reference plant for the determination of %Ndfa was the same as the test faba bean varieties. Both test and reference plants underwent similar growth conditions, but the latter received a continuous supply of inorganic-N and were not inoculated with bacteria.

### 4.4. Determination of Metabolites and Inorganic Anions

Soluble sugars, organic acids, and inorganic anions were extracted by hot water according to the procedure in [63] with some modifications [60]. Oven-dried and finely ground nodule, root, and shoot samples (~20 mg) were dissolved and boiled for 5 min in 1.5 mL of double-deionized water. They were mixed thoroughly by vortexing and immediately placed on an ice water bath for 30 min. The mixture was centrifuged, and the supernatant was transferred into a fresh Eppendorf tube. The extract was mixed with chloroform and centrifuged again. Afterwards, the supernatant was taken and filtered through strata C-18 columns (Phenomenex, Torrance, CA, USA). Finally, sugars, inorganic anions, and organic acids (OAs) were determined by isocratic ion chromatography (IC-5000 Capillary Reagent- Free I.C. System, Thermo Scientific).

### 4.5. Statistical Analyses

Data were subjected to a two-way analysis of variance (ANOVA). The significance of differences between P treatments and variety mean values for each trait were tested using a Tukey’s test HSD (honestly significant difference), where *p*-values below 0.05 were considered statistically significant, and those of *p* < 0.01 and *p* < 0.001 as highly significant. All statistical analyses and figures were conducted with R Statistical Software (Version 4.2.2., R Core Team, 2022).

## 5. Conclusions

The results have indicated that organic P (Po) supplementation imposes excellent significance on the P nutrition of faba bean plants not only in soil, but also in hydroponics. The study showed that faba bean nodules exhibited a superior capability of maintaining Pi homeostasis, as reflected by the negligible effects of P starvation on nodule P concentration compared to shoots or roots. The findings showed that nodules adapt to stress by maintaining optimum Fe, Mo, phosphate, and sulfate levels during SNF, irrespective of the nutrient status in solution. The findings further suggest that Po-supplemented plants stimulate higher OAs accumulation and exudation and improved higher P solubilization and uptake. However, the corresponding +Pi-treated plants enhanced sugars. Our results observed all negative δ^15^N signatures for the nodules, roots, and shoots of all four biological replicates of both faba bean varieties. Hence, the higher P content induced by Po treatments played a significant role in the carbon metabolism and reprogramming of metabolic processes by enhancing the mineral and carbon supply to the functioning nodules; thus, the tested hypothesis is accepted. Taken together, these results will be valuable for further efforts to elucidate the molecular mechanisms underlying faba bean nodule adaptation and symbiotic efficiencies in response to Po fertilization.

## Figures and Tables

**Table 1:** N concentration, enrichments, and P-related traits of two faba bean varieties inoculated with *Rhizobium leguminosarum* bv. *Viciae* 3841 under different phosphorus (P) treatments: 2 µM KH_2_PO_4_, 200 µM KH_2_PO_4_, and 200 µM phytic acid (denoted as −Pi, +Pi, and Po, respectively) over a period of 30 d.

Trait	Tiffany			Hiverna		
	−Pi	+Pi	Po	−Pi	+Pi	Po
Nodule N (%)	5.66 ± 0.26 ^ab^	5.96 ± 0.15 ^b^	5.73 ± 0.25 ^ab^	5.38 ± 0.18 ^ab^	5.65 ± 0.34 ^ab^	5.73 ± 0.34 ^ab^
Root N (%)	3.26 ± 0.08 ^a^	3.10 ± 0.07 ^a^	3.32 ± 0.17 ^a^	3.29 ± 0.13 ^a^	3.59 ± 0.50 ^a^	3.35 ± 0.16 ^a^
Shoot N (%)	2.71 ± 0.32 ^a^	3.15 ± 0.32 ^a^	2.70 ± 0.31 ^a^	2.54 ± 0.22 ^a^	3.02 ± 0.63 ^a^	2.43 ± 0.33 ^a^
Total N (%)	11.63 ± 0.53 ^ab^	12.21 ± 0.23 ^bc^	11.75 ± 0.51 ^abc^	11.21 ± 0.26 ^a^	12.25 ± 0.61 ^c^	11.33 ± 0.50 ^ab^
Nodule N (mg nodule^−1^)	4.03 ± 0.51 ^a^	4.45 ± 0.62 ^a^	4.06 ± 0.91 ^a^	3.14 ± 0.36 ^a^	4.56 ± 1.52 ^a^	3.80 ± 1.13 ^a^
Root N (mg root^−1^)	19.75 ± 1.78 ^c^	15.98 ± 2.11 ^abc^	14.08 ± 1.71 ^a^	18.11 ± 0.43 ^bc^	18.16 ± 0.97 ^abc^	15.70 ± 2.56 ^ab^
Shoot N (mg shoot^−1^)	31.89 ± 3.27 ^ab^	44.47 ± 3.34 ^c^	38.77 ± 6.05 ^bc^	26.34 ± 1.78 ^a^	36.88 ± 8.59 ^bc^	33.80 ± 6.93 ^abc^
Total N (mg plant^−1^)	55.67 ± 4.11 ^abc^	64.90 ± 2.82 ^c^	56.91 ± 7.66 ^abc^	47.60 ± 2.04 ^a^	59.59 ± 9.55 ^bc^	53.30 ± 10.14 ^ab^
δ^15^N nodule (‰)	−2.19 ± 0.08 ^a^	−2.10 ± 0.20 ^a^	−2.04 ± 0.25 ^a^	−2.04 ± 0.15 ^a^	−2.02 ± 0.23 ^a^	−2.46 ± 0.47 ^a^
δ^15^N root (‰)	−3.60 ± 0.56 ^a^	−3.71 ± 0.28 ^a^	−3.14 ± 0.46 ^a^	−3.22 ± 0.67 ^a^	−2.88 ± 0.43 ^a^	−2.91 ± 0.67 ^a^
δ^15^N shoot (‰)	−2.85 ± 0.38 ^a^	−2.04 ± 0.53 ^b^	−2.24 ± 0.33 ^ab^	−2.61 ± 0.28 ^ab^	−2.37 ± 0.17 ^ab^	−2.54 ± 0.22 ^ab^
%N_dfa_ shoot (%)	−60.19 ± 36.93 ^b^	−70.57 ± 40.24 ^b^	−106.19 ± 64.10 ^c^	−46.71 ± 31.76 ^a^	−100.43 ± 34.56 ^c^	−130.30 ± 52.72 ^d^
%N_dfa_ root (%)	−7.03 ± 2.23 ^b^	−22.50 ± 2.83 ^d^	8.76 ± 3.73 ^b^	2.73 ± 2.13 ^a^	4.32 ± 1.56 ^a^	15.32 ± 2.23 ^c^
Total P (mg plant^−1^)	216.68 ± 3.21 ^a^	530.07 ± 18.47 ^b^	531.84 ± 54.22 ^b^	200.62 ± 6.98 ^a^	453.30 ± 46.29 ^b^	508.47 ± 54.15 ^b^
P: N (in content)	4.02 ± 0.30 ^a^	8.02 ± 0.43 ^bc^	8.94 ± 0.37 ^d^	4.28 ± 0.13 ^a^	7.26 ± 0.39 ^b^	8.98 ± 0.82 ^cd^

Data presented are means, standard deviations, and ANOVA for varieties (V) and treatment (T) of four replicates. Different small letters indicate significant differences among the varieties and P treatment (Tukey’s test; *p* < 0.05).

## Data Availability

Data are contained within the article and Supplementary Materials.

## Supplementary Materials

The following supporting information can be downloaded at: https://www.mdpi.com/article/10.3390/plants12223888/s1.
