# Supplementary material for: Mineral and Carbon Metabolic Adjustments in Nodules of Symbiotically Grown Faba Bean (Vicia faba L.) Varieties in Response to Organic Phosphorus Supplementation
Source: Plants (Basel). 2023 Nov 17;12(22):3888. doi: 10.3390/plants12223888 (PMC10675292; doi:10.3390/plants12223888)
Supplement: Supplementary file 1 [file plants-12-03888-s001.zip › plants-2693071-supplementary.pdf]

Supplementary Materials

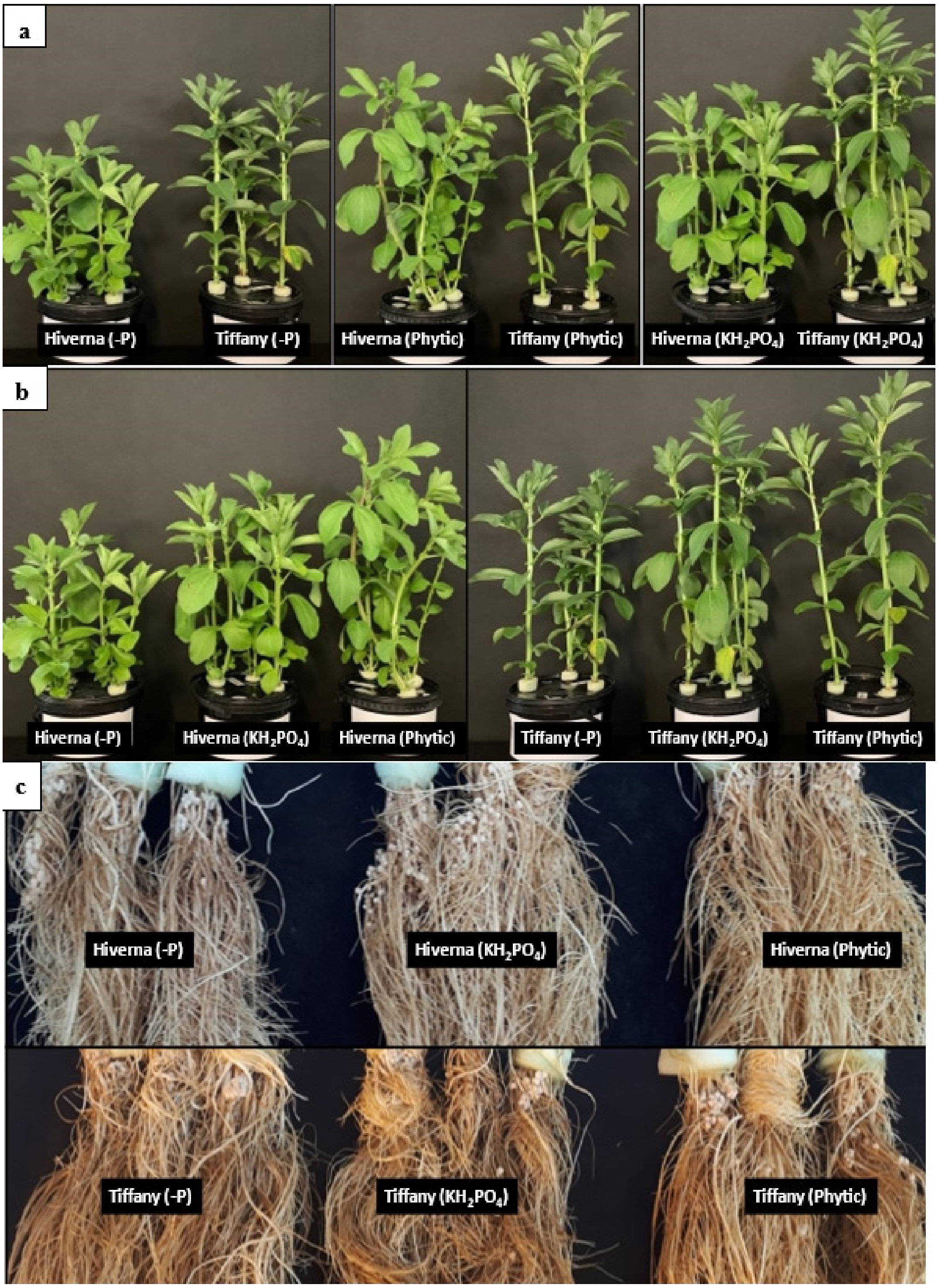

Figure S1. Photographic representation of the differences between (**a**) varieties (**b**) treatments and (**c**) nodulation of two faba bean varieties in response to low-KH_2PO_4 (-Pi), adequate-KH_2PO_4 (+Pi) and phytic acid (Po), inoculated with *Rhizobium leguminosarum* bv. *viciae* 3841 and grown in a hydroponic experiment for 30 days.

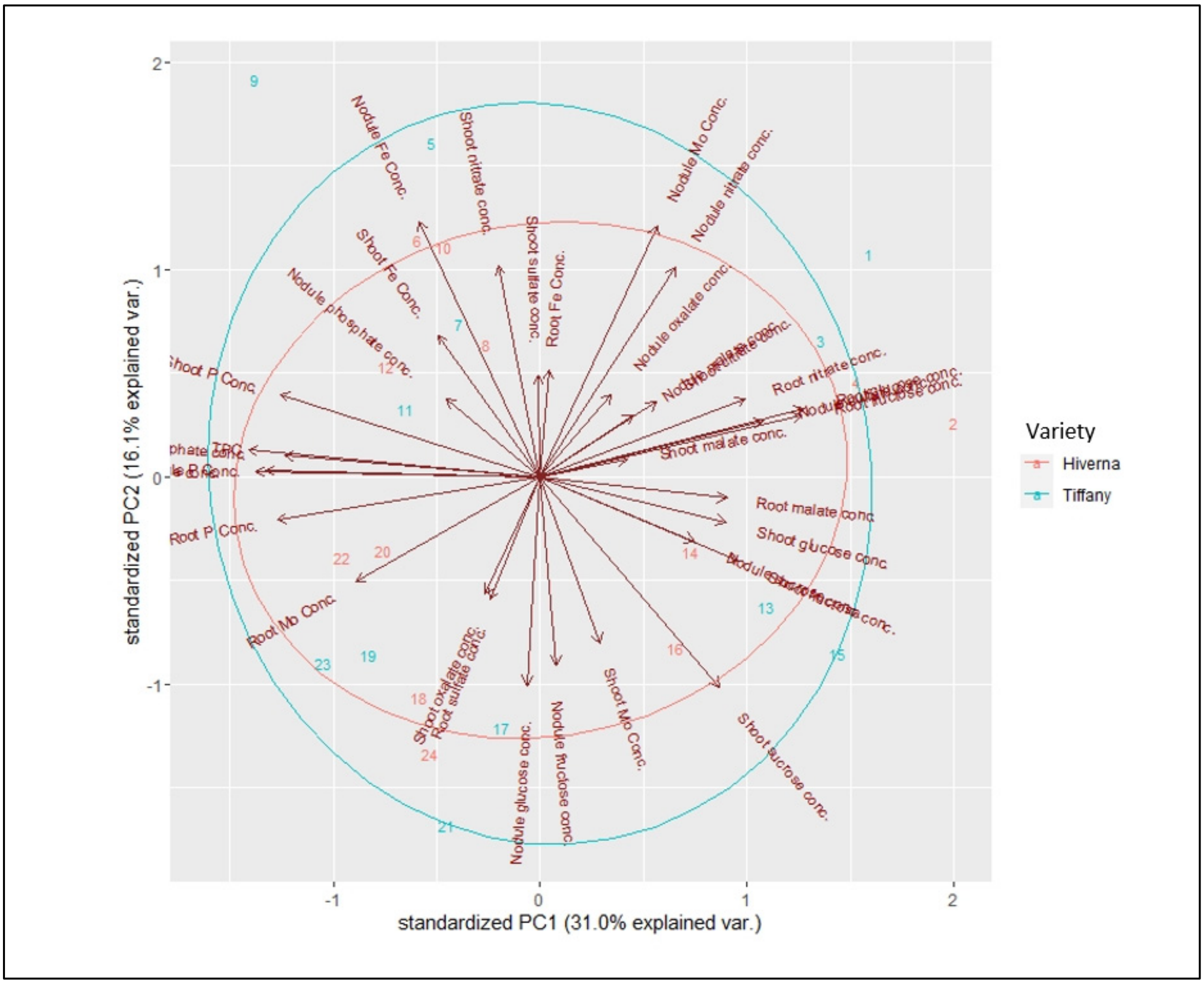

Figure S2. Principal component analysis (PCA) of individual traits measured in tissues of Tiffany and Hiverna plants supplied with low-KH_2PO_4 (−Pi), adequate-KH_2PO_4 (+Pi) and phytic acid (Po), inoculated with Rhizobium leguminosarum bv. viciae 3841 and grown in a hydroponic experiment for 30 days. PCA biplot of the treatment-variable metabolite correlations, where the angle, length and direction of the lines connecting the variable parameters indicate the strength of a positive or negative relationship.

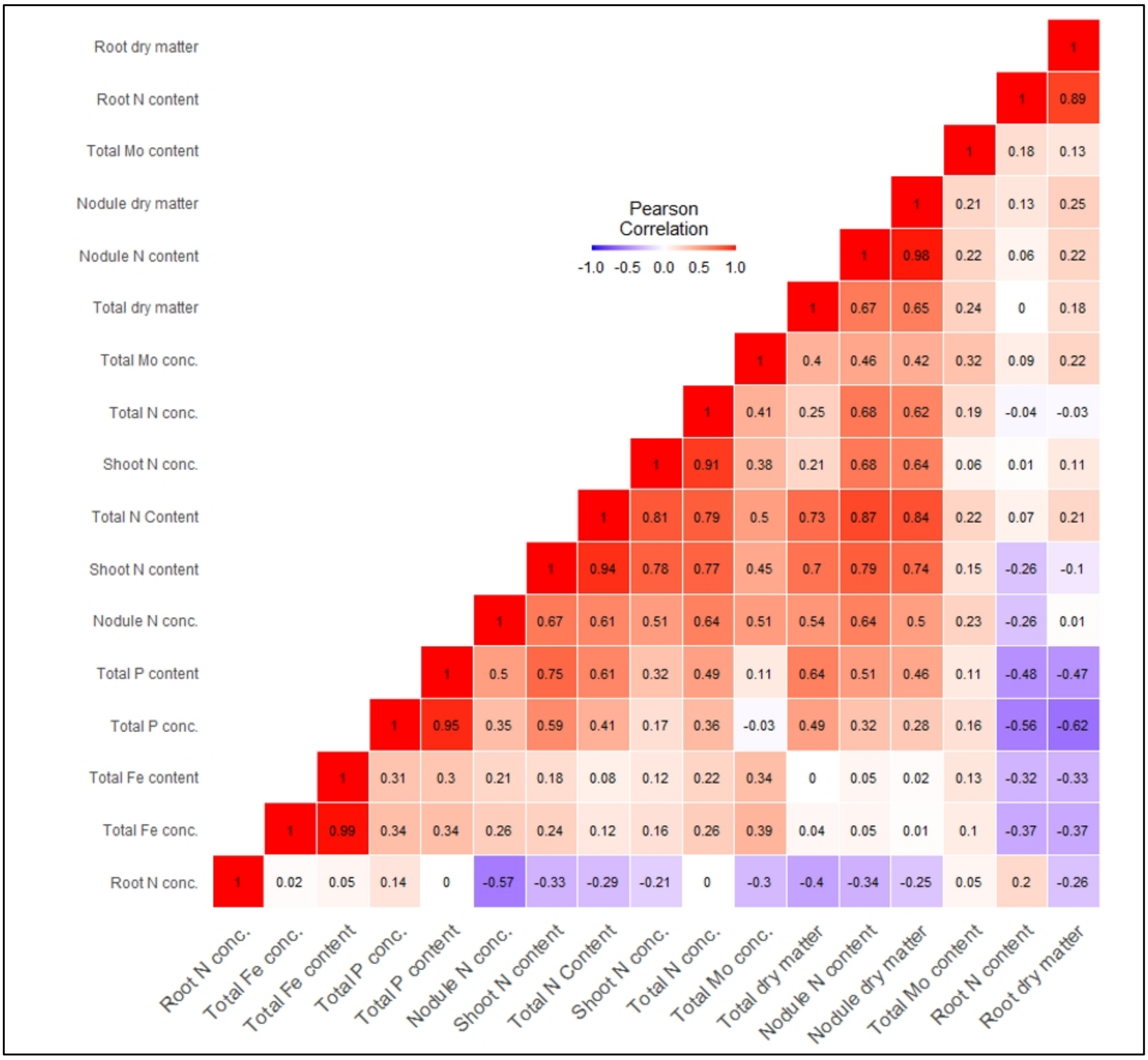

Figure S3. Heat map of correlations of 17 traits associated with plant biomass, nutrients (P, N, Fe and Mo) concentration/content of Tiffany and Hiverna varieties fertilized with low- KH_2PO_4 (−Pi), adequate-KH_2PO_4 (+Pi) and phytic acid (Po), inoculated with Rhizobium leguminosarum bv. viciae 3841 and grown in a hydroponic experiment for 30 days. Created following Pearson's r_s-values. Colour, values and square denote Pearson's ranks correlation r between traits, with four biological replicates.
